# The genetic and environmental effects on school grades in late childhood and adolescence

**DOI:** 10.1371/journal.pone.0225946

**Published:** 2019-12-31

**Authors:** Eike Friederike Eifler, Alexandra Starr, Rainer Riemann

**Affiliations:** Department of Psychology, Bielefeld University, Bielefeld, Germany; McMaster University, CANADA

## Abstract

As academic achievement can have a major impact on the development of social inequalities we set out to explore how performance differences arise. Using data of the German twin study TwinLife, genetic and environmental effects on school grades in mathematics, German and the grade point average in two age cohorts (11 and 17 years old) were identified. Structural equation modelling on the data of 432 monozygotic and 529 dizygotic twin pairs as well as 317 siblings of the twins showed substantial genetic effects (up to 62%) in both cohorts on all three variables. Next to genetic influences, the twin-specific environment as well as non-shared environmental influences were found to explain the interindividual differences in mathematics and German as well as the grade point average. A cohort effect showing itself in higher heritability in the older cohort was found for mathematics and the grade point average but not for German. Moreover, we compared twins who were assigned to the same classroom to those twins who were assigned to different classrooms and found lower effects of the twin-specific shared environment in the latter group. Our study thereby contributes to the understanding of the etiology of interindividual differences in academic achievement in the numeracy and literacy domain in two age cohorts.

## Introduction

Academic achievement is an important predictor of social success. It is connected to people’s job career, their socioeconomic status and psychological health [1–3] and can therefore have a major impact on the development of social inequalities.

Previous research has identified several cognitive factors that correlate with academic achievement. Accordingly, a strong relationship between academic achievement and IQ has been found more than once [3–5]. Moreover, working memory was confirmed to relate to academic achievement [4, 5]. Next to IQ and working memory, there also non-cognitive factors that are substantially correlated with academic achievement. These factors include the personality trait conscientiousness, the academic self-concept of students, their self-esteem, confidence, and their motivation [6–12]. Additionally, there are several factors usually considered as external that influence academic achievement. They include the type of school, the class climate, and the teacher-student relationship, the teachers themselves, the feedback they give as well as their teaching strategy, supportive parenting and socioeconomic status [10, 13–15].

Taken together, these results show that academic achievement is interconnected with a number of constructs which are all influenced by both, genes and the environment to varying degrees [16–20]. In order to gain a deeper understanding about its etiology, behavioral genetic studies have analyzed academic achievement and tried to determine how much of its variance can be attributed to genes and the environment. The study design which is most commonly used in behavioral genetics is the Classical Twin Design (CTD; [21]). In these studies, the covariance of monozygotic (MZ) twins who are genetically identical is compared to the covariance of dizygotic (DZ) twins who share half of their genes on average. By comparing the covariance of MZ and DZ twins, one can estimate the ratio of genetic variance to phenotypic variance. Structural Equation Modelling (SEM) allows the decomposition of the observed variance into genetic and environmental components, so the relative contributions of genes and the environment can be assessed.

The genetic variance can be differentiated into additive and non-additive genetic variance. Additive genetic effects (*a*^2^) describe the cumulative effects of single alleles that influence a trait in a polygenic manner. Non-additive genetic effects (*d*^2^, *i*^2^) describe the interaction of genes at a gene locus (dominance, *d*^2^) or at distinct gene loci (epistasis, *i*^2^). The environmental effects can also be differentiated into two parts, namely shared environmental effects (*c*^2^) and non-shared/specific environmental effects (*e*^2^). Shared environmental effects describe environmental stimuli that affect both twins in the same way. They therefore act to make the twins akin to each other. To the contrary, non-shared environmental effects describe environmental stimuli that affect the twins individually. They hence act to emphasis differences between twins, making them less similar to each other. Non-shared environmental effects also include error of measurement if this is not controlled for.

It is important to emphasize that the distinction between shared and non-shared effects is based on the effect they have on the similarity of family members. Not every objectively-shared event that twins experience together shows itself in a shared environmental effect. Instead, it is possible that an identical event has a unique effect on each of the twins and is thus a source of non-shared environmental variance. It is usually unclear a priori whether an event has shared or non-shared environmental effects.

Research on the heritability of academic achievement has yielded varying estimates of additive genetic effects. Johnson et al. analyzed parent and teacher ratings of general school achievement in 11 year olds and found additive genetic effects of 47% as well as substantial shared (24%) and non-shared environmental effects (29%) [22]. Additive genetic effects were also found to be the most important contributor to interindividual differences in the grade point average of adolescent sibling pairs living in the same household (*a*^2^ = 66.9%; *c*^2^ = 0.2%; *e*^2^ = 32.9%; [23]). Bartels et al. used the results of standardized exams at the end of primary school to find additive genetic effects of 57% shared environmental effects of 27% and non-shared environmental effect of 16% [24]. Heritability estimates of 72% (*c*^2^ = 14%, *e*^2^ = 14%) were found for interindividual differences in a standardized test of academic achievement in 17 year old twins in their final year of education [25]. The variability of results was also found in a meta-analysis on a total of 5330 MZ and 7084 DZ twin pairs with the heritability of educational achievement ranging from 27%-57% (*c*^2^ = 27%-57%; *e*^2^ = 8%-67%; [26]. Overall, however, the authors found educational achievement to be highly heritable while shared environmental effects only have a small effect on interindividual differences.

One possible explanation for the opposing results found in the literature might be the presence of age effects. An increase of additive genetic effects with age has already been observed for several constructs like intelligence [17]. Morris et al. found an increase in the heritability of educational attainment [27]. While additive genetic effects were found to account for 47.3% of variance in 11 year olds, their influence on interindividual differences increased to 57.6% in 14 year olds and 61.1% in 16 year olds.

Next to research on the heritability of educational achievement in various age groups, scientist have taken a look at genetic effects for distinct domains of education. Early vocabulary ability of twins in elementary school was found to be moderately heritable (*a*^2^ = 29%-49%; [28]). Using the data of the German twin study CoSMoS [29], Gottschling et al. studied the heritability of grades in German and mathematics for 13 year olds separately [30]. They found a slightly higher additive genetic effect for the performance in mathematics (44%) compared to the performance in German (38%). Similarly, de Zeuuw et al., compared the additive genetic effects in two domains of educational achievement in primary school. However, no differences were found between the heritability of arithmetic (a^2^ = 68%; c^2^ = 5%, e^2^ = 27%) and language (a^2^ = 67, c^2^ = 10%, e^2^ = 23%; [31]). Grasby et al. did not only consider distinct domains of academic achievement but also included four age groups in an Australian sample [32]. Their results confirmed similarly-high influences of genetic effects on numeracy performance (61–79%) and language performance (39–78%) throughout all age groups included in the study.

All of these results indicate that genetic—as well as environmental influences—are important when it comes to academic achievement. However, the results are not clear-cut. Varying heritability estimates for educational achievement can be found throughout the literature. Moreover, the comparison of different age groups and educational domains has not yielded straightforward results as estimates of heritability vary considerably across the studies. This way, no conclusive statement can be reached about whether age and domain effects truly exist.

Research on the separation of twins in different classrooms has yielded inconsistent results. Coventry et al. found no differences in literacy performance between twins assigned to the same versus different classrooms [33]. Similarly, educating twins in in the same versus different classrooms did not have an effect on their performance in the Dutch national academic achievement test at the end of primary school [34]. Oppositely, effects of classroom separation were found in early literacy achievement in kindergarten by Byrne et al. [35] and at age 7 by Tully et al. [36]. Separated MZ and DZ twins had lower reading scores than twins who attended the same classroom. Lower arithmetic as well as lower language scores were also found by Webbink et al. for twins in the first years of school [37]. However, by age 12 these differences were not significant anymore, implying that there are not long-lasting effects of classroom separation. Contrarily, White et al. found small effects of classroom separation on school achievement at age 12 and 16, while no differences between separated and non-separated twins were found at ages 7 to 10 [38].

Altogether, these results do not draw a clear picture on whether separating twins truly affects their school performance. The existence of a classroom separation effect can thereby not be confirmed, nor discredited.

### Overview of the current study

The current study set out to gain a deeper understanding of the etiology of academic achievement in two domains in two cohorts while controlling for the high environmental similarity of MZ twins and by using the type of measurement that reflects academic achievement most accurately. As the grades assigned on official school reports tend to differ significantly from self-reported grades [39], we used actual up to date grades as they are assigned on school reports to ensure that the construct of academic achievement was represented as accurate as possible. Moreover, the siblings of the twins were included in the analysis to overcome some of the biases of the simple CTD that arise through its restrictive assumptions. The incorporation of sibling data allows the separation of shared environmental effects into two parts. The sibling shared environmental effects (*cs*^2^) are common to all people included in the design, namely both twins as well as their sibling. The twin-shared environmental effects (*ct*^2^), however, are only common to the twins. Thereby, it was possible to ensure more accurate heritability estimates [40, 41]. Domain-specific analyses were conducted to compare heritability estimates. Achievement in the numeracy domain were represented by the grade in mathematics; achievement in the literacy domain were represented by the grade in German. Additionally the grade point average (GPA), calculated by averaging all grades listed on the current school report was analyzed. Two cohorts were used to uncover cohort effects in the different domains. Students’ performance at the age of 11 was compared with the performance of 17 year olds.

Moreover, in this study we compared the distribution of variance components of the school grades of twins who were assigned the same classroom to those twins who were assigned to different classrooms. In the German school system, being assigned to the same classroom means that children have identical timetables. Twins who were assigned to the same classroom therefore not only experienced the same class climate but were also taught and graded by the same teachers. As mentioned before, external factors like the class climate, the teacher-student relationship and the teaching strategy used by the teacher influence academic achievement [13]. Using the data of twins as well as their siblings made it possible to examine classroom effects and compare environmental variance components to gain insight into the impact of classroom related factors.

## Method

### Participants and design

Participants were extracted from the first wave of TwinLife; a genetically informative, longitudinal study about the development of social inequality [42]. This study received ethical approval from the German Psychological Association. Oral consent was obtained and recorded as part of the study procedure during the household interviews. If participants were below legal age, at least one parent or guardian at to give consent.

Only participants who were attending school at the point of the face-to-face interview could be considered as up-to-date school grades were indispensable for the work of this paper. The sample therefore only includes the twins of two of the four TwinLife cohorts: The twins in cohort C11 were 11 years old (*M* = 10.99; *SD* = 0.33) at the point of the data collection whereas the twins in the cohort C17 were 17 years old (*M* = 16.98; *SD* = 0.34) when the data collection took place. Zygosity of the twins was determined using questionnaire responses as well as saliva tests (for more detailed information, please take see Lenau and Hahn [43]). Moreover, non-twin siblings who were attending school at the point of the data collection were included in the analysis. After excluding participants for which a current school report was unavailable, the final sample consisted of 107 female MZ twin pairs, 110 male MZ twin pairs, 168 female DZ twin pairs, and 149 male DZ twin pairs in C11 as well as 124 female MZ twin pairs, 91 male MZ twin pairs, 123 female DZ twin pairs, and 89 male DZ twin pairs in C17. Furthermore, 147 female non-twin siblings as well as 170 male non-twin sibling were included in the sample. The age of the non-twin siblings ranged from 7 to 28 with a mean of 14.90 (SD = 3.90).

### Materials

Academic achievement was surveyed within TwinLife by taking photographs of the current school reports for the twins and non-twin siblings who were attending school by the time of the first study wave. As the school reports alter depending on the type of school and the federal state of Germany, the information extracted from the school report photographs was encoded into a consistent and comparable format (see Mattheus et al. [44] for an overview). Furthermore, an interview was conducted to assess various factors associated with school attendance like whether the twins were assigned to the same or different classrooms to thereby gain a more detailed overview of the course of education.

### Procedure

As part of the first face to face interview of TwinLife, a module which covered questions about the course of education and career advancement was implemented. Participants of or over the age of 16 were asked whether they had ever repeated or skipped a grade. If they affirmed this they were asked to indicate which grade they skipped or repeated.

Moreover, a school report module was carried out in the course of the same face to face interview. Those participants of or over the age of 18 who were still attending school were asked if their current school report could be photographed by the interviewer. If the participants agreed, a photograph of all pages of the current school report was taken. For participants under the age of 18, their parents were asked if a photograph of the child’s current school report could be taken.

### Analysis

**Fig 1:**
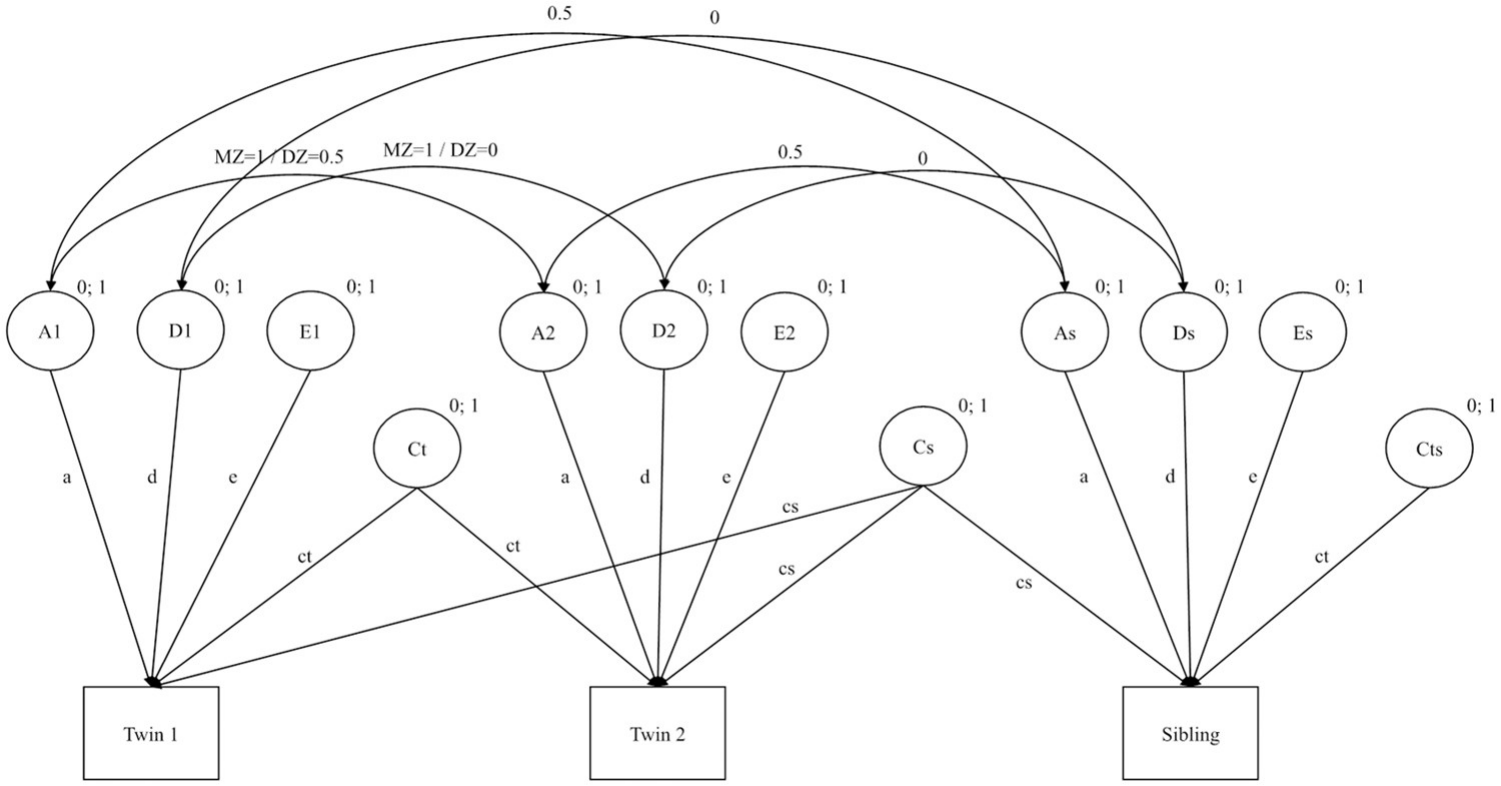
Twin sibling model for monozygotic and dizygotic twins. a = additive genetic effects; d = non-additive genetic effects; e = non-shared environmental effects; ct = twin-specific shared environmental effects; cs = shared environmental effects; cts = sibling-specific environment.

In order to control for data distortion, regression was implemented using SPSS to partial out sex, age, and type of school of the grades in German and mathematics as well as the GPA. The subsequent analyses were based on the non-standardized residuals of the three grade variables [45]. Structural equation modelling was used to ascertain the variance components of school grades by using of the statistical software AMOS (Version 23; [46]), working with the full information maximum likelihood algorithm (FIML; [47]) to handle missing data. The four group twin non-twin sibling model (zygosity by age cohort) that was fitted to the data allowed for the decomposition of the variance of the school grades into various genetic and environmental components namely additive genetic effects (a^2^), non-additive genetic effects (*d*^2^), shared environmental effects that are further partitioned in twin-shared effects (*ct*^2^) as well as sibling shared environmental effects (*cs*^2^), and non-shared environmental effects (*e*^2^) that are confounded with measurement error (see Fig 1). The χ2-difference test for nested models was used to assess whether more parsimonious models led to significant decline in model fit (see, e.g. [48]). Non-significant parameters were excluded and more parsimonious models were used if the decline in model fit was not significant. Furthermore, the comparative fit index (CFI; [49]) for which values close to 1 indicate a good fit, the root mean square of approximation (RMSEA; [50]) for which values close to 0 indicate a good fit, and the Akaike information criterion (AIC; [51, 52]) for which smaller values indicate a good fit were used as goodness-of-fit indices and for the comparison of non-nested models.

## Results

### Descriptive statistics

From the school reports, the grade point average (GPA) was calculated as the mean of all depicted grades for each student. Table 1 shows the means and standard deviations of the grades in mathematics and German as well as the GPA for the twins in both cohorts and the siblings. The variables were normally distributed with good grades being more frequent.

**Table 1:** Sample description.

		*M*	*SD*
C11	Mathematics	2.53	0.93
	German	2.51	0.84
	GPA	2.20	0.58
C17	Mathematics	2.72	1.05
	German	2.70	0.90
	GPA	2.42	0.64
Siblings	Mathematics	2.72	1.02
	German	2.65	0.92
	GPA	2.43	0.64

Note. C11 = 11 year old students; C17 = 17 year old students; *M* = mean; *SD* = standard deviation

### Genetic modelling

Intra class correlations were calculated for all three residualized grade variables in both cohorts and for MZ and DZ twins, as well as for the twins and their siblings (see Table 2). In both cohort MZ twins show stronger correlations in all three grade variables than DZ twins suggesting that additive genetic as well as shared environmental effects would be present. Correlations between the twins and their siblings were substantially lower in both cohorts and for all grade variables. Moreover, it can be seen that the correlations for all grade variables were found to be somewhat higher for the twins in C11 than for those in C17, thus smaller for the older students than for younger ones.

**Table 2:** Intra class correlations based on non-standardized residuals.

			*N*	*R*	95% C.I.	*p*
C11	Mathematics	MZ twins	203	.64	[.55–.72]	.00**
		DZ twins	284	.46	[.36–.55]	.00**
		T1 & sibling	132	.08	[-.09–.25]	.18
		T2 & sibling	137	.19	[.02–.35]	.01*
	German	MZ twins	202	.72	[.65–.78]	.00**
		DZ twins	282	.50	[.41–.59]	.00**
		T1 & sibling	129	.11	[-.06–.28]	.10
		T2 & sibling	134	.27	[.11–.42]	.00**
	GPA	MZ twins	205	.85	[.81–.89]	.00**
		DZ twins	289	.63	[.55–.69]	.00**
		T1 & sibling	135	.23	[.06–.38]	.00**
		T2 & sibling	139	.27	[.11–.42]	.00**
C17	Mathematics	MZ twins	213	.60	[.50–.68]	.00**
		DZ twins	207	.28	[.15–.40]	.00**
		T1 & sibling	94	.12	[-.08–.31]	.12
		T2 & sibling	99	.14	[-.05–.33]	.08
	German	MZ twins	213	.56	[.45–.64]	.00**
		DZ twins	205	.36	[.24–.47]	.00**
		T1 & sibling	94	.14	[-.06–.33]	.09
		T2 & sibling	99	.01	[-.19–.21]	.46
	GPA	MZ twins	214	.77	[.71–.82]	.00**
		DZ twins	210	.43	[.31–.54]	.00**
		T1 & sibling	94	.25	[.05–.43]	.01*
		T2 & sibling	100	.21	[-.08–.31]	.11

Note. C11 = 11 year old students; C17 = 17 year old students; GPA = grade point average; MZ = monozygotic; DZ = dizygotic; T1 = first-born twin; T2 = second-born twin; *r* = Pearson correlation; C.I. = confidence interval;

** = *p* < .01 bilateral significance;

* = *p* < .05 bilateral significance

Since d^2^ and cs^2^ could not be estimated in the same model an ADCtE model (*cs* = 0) which allowed the estimation of non-additive genetic effects rather than the estimation of environmental effects shared by all siblings was chosen as baseline model for the all three grade variables because it yielded a better fit than the ACsCtE model (*d* = 0; see S2 Table for fit statistics). Using the χ2-difference test the baseline models were reduced by testing whether a more parsimonious model resulted in a significantly poorer model fit (see S1 Table for detailed model comparisons). For all cases a model including additive genetic effects (*a*), twin-shared environmental (*ct*) as well as non-shared environmental effects (*e*) was found to be the best compromise between parsimony and model fit, confirming an ACtE model (*d* = *cs* = 0; see S1 and S2 Tables).

#### Cohort effects

A four group ACtE model (*d* = *cs* = 0) allowing for cohort specific parameter estimates was implemented for each of the three grade variables to test for differences in the etiology of school grades in the two age groups. This model was then used as baseline model and compared to the more parsimonious ACtE model without cohort differentiation (C.D.). The comparison of the model with and without C.D. was performed to test whether possible differences in variance estimates between cohorts were significant. For all three grade variables the ACtE model without C.D. resulted in a significantly worse fit, indicating that parameter estimates cannot be generalized across the two age groups and that differences between cohorts were significant (see S3 Table for detailed model comparisons and S4 Table for fit statistics).

In a next step it was tested whether the ACtE model with C.D. could be reduced to a more parsimonious model without yielding a significantly worse fit. In doing so, an AE model (*d* = *cs* = *ct* = 0) was found for the students in C17 on the grade variable of mathematics. The most parsimonious models for each grade variable in the two age groups as well as their standardized variance components are shown in Table 3. The heritability of mathematics was found to be higher in C17 (*a*^2^_C17_ = .58) than in C11 (*a*^2^_C11_ = .34), confirming the anticipated cohort effect for this grade variable. Moreover, there was no effect of the shared environment in the C17, whereas it explained 33% of the variance in C11. The cohort effects found for mathematics could, however, not be confirmed by the grade in German. Here, the variance attributed to genetics was smaller for the students in C17 (*a*^2^_C17_ = .34) compared to those in C11 (*a*^2^_C11_ = .48). Almost no difference was found between the shared environmental effects in the two cohorts for this grade variable. However, the effect of the non-shared environment was found to be larger in C17 (*e*^2^_C17_ = .43) compared to C11 (*e*^2^_C11_ = .24). Finally, the results for the GPA indicated a much higher heritability for the older than for younger students, *a*^2^_C17_ = .62 and *a*^2^_C11_ = .47, respectively. A big difference could also be seen for the shared environmental effect, with it being smaller in C17 (*ct*^2^_C17_ = .16) compared to C11 (*ct*^2^_C11_ = .40).

**Table 3:** Standardized variance components derived from the best fitting, most parsimonious model.

		*a*^2^	*ct*^2^	*e*^2^
Mathematics	C11	.34	.33	.33
	C17	.58	-	.42
German	C11	.48	.28	.24
	C17	.34	.23	.43
GPA	C11	.47	.40	.13
	C17	.62	.16	.22

Note. *a*^2^ = additive genetic effects; *ct*^2^ = twin-shared environmental effects; *e*^2^ = non-shared environmental effects (including measurement error)

#### Classroom effects

For C11 we compared twins who were attending the same classroom to twins who were assigned to different classrooms. Having been assigned the same classroom meant that the twins were taught and graded by the same teacher, thus increasing their environmental similarity. Twins for which there was no information on whether they shared a classroom or not were excluded leaving a final sample of 103 female MZ twin pairs, 102 male MZ twin pairs, 130 female DZ twin pairs, 141 male DZ twin pairs, 78 female non-twin siblings, and 84 male non-twin siblings. The age of the non-twin siblings ranged from 7 to 21 with a mean of 13.37 (SD = 2.85).

A four group ACtE model (*d* = *cs* = 0) allowing for group differentiation (G.D.) was implemented for each of the three grade variables to test whether there were significant differences in the etiology of school grades in twins who were assigned to the same classroom versus those twins who were assigned to different classrooms. For all three grade variables the ACtE model allowing for G.D. was found to be the best fitting model indicating that differences between groups were significant. For all three grade variables additive genetic variance (*a*^2^) as well as the non-shared environmental effects (e^2^) were found to be bigger for twins who were assigned to different classrooms compared to twins who were assigned to the same classroom. The twin-shared environmental effects (ct^2^), however, were found to be smaller for the former group. Table 4 depicts the best fitting model for each grade variable as well as their standardized variance components.

**Table 4:** Standardized variance components derived from the best fitting, most parsimonious model.

		*a*^2^	*ct*^2^	*e*^2^
Mathematics	Same class	.35	.35	.30
	Different classes	.38	.22	.40
German	Same class	.42	.37	.21
	Different classes	.65	.07	.28
GPA	Same class	.43	.48	.09
	Different classes	.60	.22	.18

Note. *a*^2^ = additive genetic effects; *ct*^2^ = twin-shared environmental effects; *e*^2^ = non-shared environmental effects (including measurement error)

Using this model as baseline model it was further assess whether more parsimonious models would yield a significantly worse fit. In doing so, a rather unclear picture across the three grade variables unfolded: For the GPA the ACtE model allowing for differences between groups was kept as it was found to be the best compromise between model fit and parsimony. However, for both mathematics and German, the χ2-difference test yielded two models that did not yield a significantly worse fit while being more parsimonious. On one hand, the ACtE model without G.D. was found to not reduce the model fit significantly. On the other hand, a model allowing for G.D. was found. This model included additive genetic effects (*a*^2^), twin-shared environmental (*ct*^2^) effects as well as non-shared environmental effects (*e*^2^) for twins who were assigned to the same classroom. For twins who were assigned to different classrooms, however, environmental effects shared by twins (*ct*^2^) were negligible. A detailed overview of model comparisons and fit statistics can be found in the supporting information of this paper (S5 and S6 Tables, respectively).

## Discussion

In this study, a twin sibling design was used to gain deeper insight into the etiology of school grades. Much of the variance of all three grade variables was explained by additive genetic effects, while twin-shared environmental effects as well as non-shared environmental effect explained the remaining variance. Heritability was higher in the older cohort (C17) for mathematics and the GPA but not for German.

It is important to mention that this does not indicate that school grades are unchangeable in older students. Rather, the results describe how interindividual differences might arise. Genetic predispositions set the developmental frame on which’s basis the peculiarity of a trait is shaped by environmental influences and gene-environment interplay. Additive genetic effects (*a*^2^) as well as twin-shared environmental effects (*ct*^2^) in the absence of effects of the environment shared by all siblings (*cs*^2^) imply that grades depend on the children’s partially heritable abilities instead of on the family status, income or other factors shared by all siblings; emphasizing that grades in German schools largely reflect individual achievement. This finding indicates that, contrarily to common socioeconomic explanations, shared environmental effects such as family background and parenting are not very important to student’s academic achievement. In contrast, the twin-shared environmental was found to play a major role in explaining interindividual differences in school performance.

Although the comparison between twins who were assigned to the same classroom and those twins who were assigned to different classrooms yielded somewhat unclear results and ask for more elaborate research on this topic, it seems to be a good starting point for the explanation of what the twin-shared environment reflects. The findings demonstrate the significance of classroom specific influences that are partly responsible for interindividual differences in academic achievement.

The heritability estimates we found in our study are generally consistent with that of previous studies [24, 22]. Furthermore, a cohort effect could be confirmed for the grade mathematics as well as for the GPA. However, we did not find the heritability of the 17-year old students to be bigger than that of the 11-year old students for German. One reason for the reverse cohort effect found for German could be the curriculum change that occurs in this subject: In primary school basic competences like reading, spelling, punctuation and grammar are acquired. These skills follow a strict schema that allows for unambiguous judgement and are more strongly related to fluid intelligence [53]. In higher grades the curriculum changes to more artistic tasks like creative writing or the interpretation of literature that require not only the basic rules of punctuation and grammar but also eloquence. These performances follow a rather unclear grading system that is susceptible to biases. This shift from clear requirements to a rather ambiguous grading system might explain that the non-shared environmental influences (including measurement error) of the grade in German in C17 are heightened on the cost of the heritability estimate.

Moreover, in this study we took a look at dominance effects (*d*^2^) and found them to be unimportant. The disaggregation of shared environmental effects (*c*^2^), however, yielded a unique insight into the ways in which the environment brings about interindividual differences in academic achievement. In opposition to conventional socioeconomic explanations of interindividual differences, sibling shared environmental effects (*cs*^2^) were found to be negligible. This result scrutinizes the common assumption that environmental influences shared by all children of a family affect academic achievement [54, 55]. Thereupon, factors like parenting and socioeconomic status of both the students and the schools are not found to influence all children in a family in the same way. In contrast, twin-shared environmental effects (*ct*^2^) were found to be of great importance. These effects partly reflect classroom specific influences as can be seen in the results of the comparison between twins who were assigned to the same classroom and twins who were assigned to different classrooms.

The decomposition of variance for twins who were assigned to the same classroom was comparable to the variance decomposition in C11 found by us using the twin-sibling model (see Table 3). The distribution of variance for twins who were assigned to different classrooms, however, was quite different for the GPS as well as for German: The heritability was found to be much bigger for these two grade variables whereas twin-shared environmental influences were found to be reduced. For German, a more parsimonious model that did not include twin-shared environmental influences at all was therefore found to not reduce the model fit (see S5 and S6 Tables). These results point towards the significance of environmental influences of classrooms that seem to partly be responsible for interindividual differences in academic achievement. These classroom specific influences include the teachers, their teaching strategy as well as the feedback they give, the teacher-student relationship, teachers’ rater effects (like biases and lenience), and the class climate. Moreover, it should be mentioned that the non-shared environmental influences of all three grade variables are only somewhat higher for twins who were assigned to different classrooms compared to those who were assigned to the same classroom. This points towards truly individual influences as well as judgment biases that lead to differences in academic achievement. The measurement of the grades was always objective in our study, meaning that the measurements used in our analysis were not confounded by using children’s or parent’s reports on their achieved grades but the grades as they were assigned on the official school reports. However, school grades are assigned by teachers on the basis of exams and oral contributions in the classroom. In this process judgement biases come about because teachers value certain performance aspects more strongly than others. Moreover, stereotypes (e.g. gender) may have an effect on grades. Biases made by teachers on the basis of certain stereotypic assumptions mean that more variance is attributed to non-shared environmental influences in the individual subjects of mathematics and German. When averaging all grades and forming the GPA, these biases are somewhat controlled for which can be seen in the slightly smaller non-shared environmental influences for this grade variable.

### Limitations

There are several limitations restricting this study’s generalizability as well as possible distortions that could not be met by expanding the classical twin design to include the siblings of the twins. Firstly, the twin sibling design does not make it possible to investigate gene-environment interplay. Ignoring active or reactive gene-environment correlations can lead to an overestimation of the heritability as they are confounded with the genetic variance component [40, 41, 56]. Furthermore, the heritability can be underestimated in the presence of measurement error influences as the dissimilarity of MZ and DZ twins are inflated and therefore reflected in the non-shared environmental variance component.

Another limitation to this study is the rather small sample size and the wide age range of the twins’ siblings lead to a reduction of the statistical power of our findings. Moreover, it must be mentioned that the names of the teachers of the twins and siblings were unknown. This way twins, as well as siblings, could have been taught by the same teacher although they were assigned to different classrooms or even different school years. The comparison between twins who were assigned to the same classroom and those who were assigned to different classrooms helps to somewhat overcome this limitation and allows for a better understanding of shared environmental influences.

### Implications and future directions

Although the aforementioned limitations confine the generalizability and interpretability of our results to some extent, this study contributes significantly to the research on the origin of school grades. The results imply that genetic predisposition plays a major role in establishing interindividual differences in school grades. Furthermore, non-shared environmental factors play a key factor in explaining why people differ in their academic achievement. These experiences might truly be individual like having a partner and friends who are not shared with any sibling. However, they might also reflect experiences that are shared by all children of a family (e.g. parenting style) but perceived differently by each one of them. Our results thereby point towards the necessity to focus on individual aspects of young people in order to understand why they differ in their academic achievement and how they can be helped to unfold their full potential just like Stumm and Plomin suggested [57]. Lastly, our results indicate a strong influence of the environment shared by the twins but not their sibling. These twin-shared environmental effects could reflect influences of age-specific experiences, demographics, peer-groups and–in the case of twins being assigned to the same classroom–classroom specific influences.

The results of the comparison between twins who were assigned to the same classroom and those who were assigned to different classrooms are somewhat unclear. Notwithstanding, the significantly lower twin-shared environment for German in twins who were assigned to different classrooms shows that experiences made in class play a major role in constituting dissimilarities in student’s school grades. Future research should therefore analyze classroom specific aspects like class climate, teacher-student relationship and teaching strategy and their effect on the achievement of students in greater detail. Because of the rather small sample size used for this analysis, future research is encouraged to examine the comparison of twins being taught by the same teachers in the same class climate and those twins who have different timetables.

Heritability was higher in the older cohort for mathematics but not for German language. The results confirm a cohort effect for mathematics with genetics explaining 34% of its variance in C11 and 58% in C17. Contrary to the cohort effect found for mathematics, the variance of German was explained by genetics to 48% in C11 and 34% in the C17. One possible explanation for this finding is that different abilities are needed in the numeracy and literacy domain. Whereas abilities like logical thinking and complex problem solving as well as deductive and inductive reasoning are needed for numeracy performance like mathematics, literacy performance relies on abilities like creativity as well as on vocabulary and eloquence. Future explorative research should study the subjects’ matter more closely to find out the differences in grading criteria for the numeracy and literacy domain subjects and their heritability. In doing so, a comparison between different countries, their school systems and grading criteria could lead to a greater insight into their advantages and disadvantages.

Several questions remain unanswered and demand further research. Longitudinal data on a broader age range will give a better insight into the stability and change of the variance components. Furthermore, it would be interesting to examine more subjects in the numeracy and literacy domain like foreign languages as well as natural sciences. Moreover, an extended twin family design that not only includes the data of twins and siblings but also that of the twin’s parents could be used in future studies so get a deeper insight on intra-familiar effects. Additionally, this design would allow to make more confident predications about non-additive genetic variation.

### Conclusion

In this study we used a twin sibling design to get a deeper insight into the etiology of interindividual differences in school grades. Next to genetic influences, the twin-shared environment as well as non-shared environmental influences were found to explain the interindividual differences in mathematics and German as well as the GPA. A cohort effect displayed in higher heritability in older students (C17) was found for mathematics as well as the GPA but not for German. Future research is therefore encouraged to analyze the different abilities needed in order to be successful in the numeracy and literacy domain as well as their heritability.

## Supporting information

S1 Table Model comparison: χ2-difference test for models without cohort differentiation. *Note*. A = additive genetic effects; D = non-additive genetic effects; Ct = twin-shared environmental effects; E = non-shared environmental effects (including measurement error); ADCtE model = *cs* = 0; ACtE model = *d* = *cs* = 0; ADE model = *cs* = *ct* = 0; AE model = *d* = *cs* = *ct* = 0; CtE model = *a* = *d* = *cs* = 0; *p* = bilateral significance; ** = *p* < .01 bilateral significance; * = *p* < .05 bilateral significance.
(DOCX)

S2 Table Model comparison tests and fit-statistics for models without cohort differentiation. *Note*: A = additive genetic effects; D = non-additive genetic effects; Ct = twin-shared environmental effects; E = non-shared environmental effects (including measurement error); p = two-sided significance; CFI = Comparative Fit Index; RMSEA = Root Mean Square of Approximation; AIC = Akaike Information Criterion; ** = *p* < .01 bilateral significance; * = *p* < .05 bilateral significance.
(DOCX)

S3 Table Model comparison: χ2-difference test for models without cohort differentiation. *Note*. A = additive genetic effects; D = non-additive genetic effects; Ct = twin-shared environmental effects; E = non-shared environmental effects (including measurement error); ACtE model = *d* = *cs* = 0; AE model = *d* = *cs* = *ct* = 0; CtE model = *a* = *d* = *cs* = 0; C.D. = cohort differentiation; *p* = two-sided significance; ** = *p* < .01 bilateral significance; * = *p* < .05 bilateral significance.
(DOCX)

S4 Table Model comparison tests and fit-statistics for models allowing for cohort differentiation. *Note*: A = additive genetic effects; D = non-additive genetic effects; Ct = twin-shared environmental effects; E = non-shared environmental effects (including measurement error); ACtE model = *d* = *cs* = 0; AE model = *d* = *cs* = *ct* = 0; CtE model = *a* = *d* = *cs* = 0; C.D. = cohort differentiation; *p* = two-sided significance; CFI = Comparative Fit Index; RMSEA = Root Mean Square of Approximation; AIC = Akaike Information Criterion; ** = *p* < .01 bilateral significance; * = *p* < .05 bilateral significance.
(DOCX)

S5 Table Model comparison: χ2-difference test for models with differentiation between twins who were assigned to the same classroom and twins who were assigned to different classrooms. *Note*. A = additive genetic effects; D = non-additive genetic effects; Ct = twin-shared environmental effects; E = non-shared environmental effects (including measurement error); ACtE model = *d* = *cs* = 0; AE model = *d* = *cs* = *ct* = 0; CtE model = *a* = *d* = *cs* = 0; G.D. = group differentiation; sc = twins who were assigned to the same class; ds = twins who were assigned to different classes; *p* = two-sided significance; ** = *p* < .01 bilateral significance; * = *p* < .05 bilateral significance.
(DOCX)

S6 Table Model comparison tests and fit-statistics for models allowing for differentiation between twins who were assigned to the same classroom and twins who were assigned to different classrooms. *Note*: A = additive genetic effects; D = non-additive genetic effects; Ct = twin-shared environmental effects; E = non-shared environmental effects (including measurement error); ACtE model = *d* = *cs* = 0; AE model = *d* = *cs* = *ct* = 0; CtE model = *a* = *d* = *cs* = 0; G.D. = group differentiation; sc = twins who were assigned to the same class; dc = twins who were assigned to different classrooms; *p* = two-sided significance; CFI = Comparative Fit Index; RMSEA = Root Mean Square of Approximation; AIC = Akaike Information Criterion; ** = *p* < .01 bilateral significance; * = *p* < .05 bilateral significance.
(DOCX)

S7 Table Standardized variance components derived from the best fitting model as well as from more parsimonious models. *Note*. *a*^2^ = additive genetic effects; *ct*^2^ = twin-shared environmental effects; *e*^2^ = non-shared environmental effects (including measurement error); G.D. = group differentiation.
(DOCX)

S8 Table Unstandardized path estimates and 95% confidence intervals for model parameters derived from the best fitting, most parsimonious model. *Note*. *a* = additive genetic effects; *ct* = twin-shared environmental effects; *e* = non-shared environmental effects (including measurement error).
(DOCX)

S9 Table Unstandardized path estimates and 95% confidence intervals for model parameters derived from the best fitting, most parsimonious model. *Note*. *a* = additive genetic effects; *ct* = twin-shared environmental effects; *e* = non-shared environmental effects (including measurement error).
(DOCX)
