# Supplementary material for: The genetic and environmental effects on school grades in late childhood and adolescence
Source: PLoS One. 2019 Dec 31;14(12):e0225946. doi: 10.1371/journal.pone.0225946 (PMC6938312; doi:10.1371/journal.pone.0225946)
Supplement: S1 Table — Note. A = additive genetic effects; D = non-additive genetic effects; Ct = twin-shared environmental effects; E = non-shared environmental effects (including measurement error); ADCtE model = cs = 0; ACtE model = d = cs = 0; ADE model = cs = ct = 0; AE model = d = cs = ct = 0; CtE model = a = d = cs = 0; p = bilateral significance; ** = p < .01 bilateral significance; * = p < .05 bilateral significance. (DOCX) [file pone.0225946.s001.docx]

**S1 Table. Model comparison: χ2-difference test for models without cohort differentiation.**

|  | **Model** |  | **χ2** | ***df*** | ***Δ* χ2** | **Δ *df*** | ***p*** |
| --- | --- | --- | --- | --- | --- | --- | --- |
| Mathematics | ADCtE |  | 37.79 | 20 |  |  |  |
|  |  | **ACtE** | **39.51** | **21** | **1.72** | **1** | **.19** |
|  |  | ADE | 54.89 | 21 | 17.10 | 1 | .00^**^ |
|  | **ACtE** |  | **39.51** | **21** |  |  |  |
|  |  | AE | 55.85 | 22 | 16.35 | 1 | .00^**^ |
|  |  | CtE | 72.35 | 22 | 32.84 | 1 | .00^**^ |
| German | ADCtE |  | 39.80 | 20 |  |  |  |
|  |  | **ACtE** | **40.25** | **21** | **0.45** | **1** | **.50** |
|  |  | ADE | 63.87 | 21 | 24.07 | 1 | .00^**^ |
|  | **ACtE** |  | **40.25** | **21** |  |  |  |
|  |  | AE | 63.87 | 22 | 23.63 | 1 | .00^**^ |
|  |  | CtE | 76.42 | 22 | 36.18 | 1 | .00^**^ |
| GPA | ADCtE |  | 53.08 | 20 |  |  |  |
|  |  | **ACtE** | **53.17** | **21** | **0.08** | **1** | **.77** |
|  |  | ADE | 88.73 | 21 | 35.64 | 1 | .00^**^ |
|  | **ACtE** |  | **53.17** | **21** |  |  |  |
|  |  | AE | 88.73 | 22 | 35.56 | 1 | .00^**^ |
|  |  | CtE | 165.24 | 22 | 112.07 | 1 | .00^**^ |

Note. A = additive genetic effects; D = non-additive genetic effects; Ct = twin-shared environmental effects; E = non-shared environmental effects (including measurement error); ADCtE model = cs=0; ACtE model = d=cs=0; ADE model = cs=ct=0; AE model = d=cs=ct=0; CtE model = a=d=cs=0; p = bilateral significance; ** = p < .01 bilateral significance; * = p < .05 bilateral significance
