# Supplementary material for: The genetic and environmental effects on school grades in late childhood and adolescence
Source: PLoS One. 2019 Dec 31;14(12):e0225946. doi: 10.1371/journal.pone.0225946 (PMC6938312; doi:10.1371/journal.pone.0225946)
Supplement: S2 Table — Note: A = additive genetic effects; D = non-additive genetic effects; Ct = twin-shared environmental effects; E = non-shared environmental effects (including measurement error); p = two-sided significance; CFI = Comparative Fit Index; RMSEA = Root Mean Square of Approximation; AIC = Akaike Information Criterion; ** = p < .01 bilateral significance; * = p < .05 bilateral significance. (DOCX) [file pone.0225946.s002.docx]

**S2 Table. Model comparison tests and fit-statistics for models without cohort differentiation.**

|  | **Model** | **χ2** | ***df*** | ***p*** | **CFI** | **RMSEA** | **AIC** |
| --- | --- | --- | --- | --- | --- | --- | --- |
| Mathematics | ACtCsE | 39.51 | 20 | .01^*^ | .93 | .022 | 71.51 |
|  | ADCtE | 37.79 | 20 | .01^*^ | .94 | .021 | 69.79 |
|  | **ACtE** | **39.51** | **21** | **.01^*^** | **.93** | **.021** | **69.51** |
|  | ADE | 54.89 | 21 | .00^**^ | .88 | .028 | 84.89 |
|  | AE | 55.85 | 22 | .00^**^ | .88 | .027 | 83.85 |
|  | CtE | 72.35 | 22 | .00^**^ | .82 | .033 | 100.35 |
| German | ACtCsE | 40.25 | 20 | .01^*^ | .94 | .022 | 72.25 |
|  | ADCtE | 39.80 | 20 | .01^*^ | .94 | .022 | 71.80 |
|  | **ACtE** | **40.25** | **21** | **.01^*^** | **.94** | **.021** | **70.25** |
|  | ADE | 63.87 | 21 | .00^**^ | .87 | .031 | 93.87 |
|  | AE | 63.87 | 22 | .00^**^ | .87 | .030 | 91.87 |
|  | CtE | 76.42 | 22 | .00^**^ | .84 | .034 | 104.42 |
| GPA | ACtCsE | 53.17 | 20 | .00^*^ | .95 | .028 | 85.17 |
|  | ACsCtE | 53.08 | 20 | .00^**^ | .95 | .028 | 85.08 |
|  | **ACtE** | **53.17** | **21** | **.00^**^** | **.95** | **.027** | **83.17** |
|  | ADE | 88.73 | 21 | .00^**^ | .90 | .039 | 118.73 |
|  | AE | 88.73 | 22 | .00^**^ | .90 | .038 | 116.73 |
|  | CtE | 165.24 | 22 | .00^**^ | .78 | .056 | 193.24 |

*Note*: A = additive genetic effects; D = non-additive genetic effects; Ct = twin-shared environmental effects; E = non-shared environmental effects (including measurement error); *p* = two-sided significance; CFI = Comparative Fit Index; RMSEA = Root Mean Square of Approximation; AIC = Akaike Information Criterion; ** = *p* < .01 bilateral significance; * = *p* < .05 bilateral significance
