# Supplementary material for: The genetic and environmental effects on school grades in late childhood and adolescence
Source: PLoS One. 2019 Dec 31;14(12):e0225946. doi: 10.1371/journal.pone.0225946 (PMC6938312; doi:10.1371/journal.pone.0225946)
Supplement: S3 Table — Note. A = additive genetic effects; D = non-additive genetic effects; Ct = twin-shared environmental effects; E = non-shared environmental effects (including measurement error); ACtE model = d = cs = 0; AE model = d = cs = ct = 0; CtE model = a = d = cs = 0; C.D. = cohort differentiation; p = two-sided significance; ** = p < .01 bilateral significance; * = p < .05 bilateral significance. (DOCX) [file pone.0225946.s003.docx]

**S3 Table. Model comparison: χ2-difference test for models without cohort differentiation.**

|  | **Model** |  | **χ2** | ***Df*** | ***Δ* χ2** | **Δ *df*** | ***p*** |
| --- | --- | --- | --- | --- | --- | --- | --- |
| Mathematics | ACtE_with C.D._ |  | 15.46 | 18 |  |  |  |
|  |  | ACtE_without C.D._ | 39.51 | 21 | 24.05 | 3 | .00^**^ |
|  |  | **C11: ACtE; C17:AE** | **16.79** | **19** | **1.33** | **1** | **.25** |
|  |  | C11: AE; C17: ACtE | 37.34 | 19 | 21.88 | 1 | .00^**^ |
|  |  | C11: ACtE; C17: CtE | 35.38 | 19 | 19.92 | 1 | .00^**^ |
|  |  | C11: CtE; C17: ACtE | 30.93 | 19 | 15.48 | 1 | .00^**^ |
| German | **ACtE_with C.D._** |  | **15.01** | **18** |  |  |  |
|  |  | ACtE without C.D. | 40.25 | 21 | 25.24 | 3 | .00^**^ |
|  |  | C11: ACtE; C17:AE | 22.01 | 19 | 7.00 | 1 | .01^*^ |
|  |  | C11: AE; C17: ACtE | 32.76 | 19 | 17.76 | 1 | .00^**^ |
|  |  | C11: ACtE; C17: CtE | 24.86 | 19 | 9.85 | 1 | .00^**^ |
|  |  | C11: CtE; C17: ACtE | 51.48 | 19 | 36.47 | 1 | .00^**^ |
| GPA | **ACtE_with C.D._** |  | **16.10** | **18** |  |  |  |
|  |  | ACtE_without C.D._ | 53.17 | 21 | 37.07 | 3 | .00^**^ |
|  |  | C11: ACtE; C17:AE | 20.83 | 19 | 4.73 | 1 | .03^*^ |
|  |  | C11: AE; C17: ACtE | 57.41 | 19 | 41.31 | 1 | .00^**^ |
|  |  | C11: ACtE; C17: CtE | 68.21 | 19 | 52.11 | 1 | .00^**^ |
|  |  | C11: CtE; C17: ACtE | 84.77 | 19 | 68.68 | 1 | .00^**^ |

Note. A = additive genetic effects; D = non-additive genetic effects; Ct = twin-shared environmental effects; E = non-shared environmental effects (including measurement error); ACtE model = d=cs=0; AE model = d=cs=ct=0; CtE model = a=d=cs=0; C.D. = cohort differentiation; p = two-sided significance; ** = p < .01 bilateral significance; * = p < .05 bilateral significance
