# Supplementary material for: The genetic and environmental effects on school grades in late childhood and adolescence
Source: PLoS One. 2019 Dec 31;14(12):e0225946. doi: 10.1371/journal.pone.0225946 (PMC6938312; doi:10.1371/journal.pone.0225946)
Supplement: S4 Table — Note: A = additive genetic effects; D = non-additive genetic effects; Ct = twin-shared environmental effects; E = non-shared environmental effects (including measurement error); ACtE model = d = cs = 0; AE model = d = cs = ct = 0; CtE model = a = d = cs = 0; C.D. = cohort differentiation; p = two-sided significance; CFI = Comparative Fit Index; RMSEA = Root Mean Square of Approximation; AIC = Akaike Information Criterion; ** = p < .01 bilateral significance; * = p < .05 bilateral significance. (DOCX) [file pone.0225946.s004.docx]

**S4 Table. Model comparison tests and fit-statistics for models allowing for cohort differentiation.**

|  | **Model** | **χ2** | ***df*** | ***P*** | **CFI** | **RMSEA** | **AIC** |
| --- | --- | --- | --- | --- | --- | --- | --- |
| Mathematics | ACtE_with C.D._ | 15.46 | 18 | .63 | 1.00 | .000 | 51.46 |
|  | ACtE_without C.D._ | 39.51 | 21 | .01^*^ | .93 | .021 | 69.51 |
|  | **C11: ACtE; C17:AE** | **16.79** | **19** | **.60** | **1.00** | **.000** | **50.79** |
|  | C11: AE; C17: ACtE | 37.34 | 19 | .01^*^ | .93 | .021 | 71.34 |
|  | C11: ACtE; C17: CtE | 35.38 | 19 | .01^*^ | .94 | .020 | 69.38 |
|  | C11: CtE; C17: ACtE | 30.93 | 19 | .04^*^ | .96 | .017 | 64.93 |
| German | **ACtE_with C.D._** | **15.01** | **18** | **.66** | **1.00** | **.000** | **51.01** |
|  | ACtE_without C.D._ | 40.25 | 21 | .01^*^ | .94 | .021 | 70.25 |
|  | C11: ACtE; C17:AE | 22.01 | 19 | .28 | .99 | .009 | 56.01 |
|  | C11: AE; C17: ACtE | 32.76 | 19 | .03^*^ | .96 | .019 | 66.76 |
|  | C11: ACtE; C17: CtE | 24.86 | 19 | .17 | .98 | .012 | 58.86 |
|  | C11: CtE; C17: ACtE | 51.48 | 19 | .00^**^ | .90 | .029 | 85.48 |
| GPA | **ACtE_with C.D._** | **16.10** | **18** | **.59** | **1.00** | **.000** | **52.10** |
|  | ACtE_without C.D._ | 53.17 | 21 | .00^**^ | .95 | .027 | 83.17 |
|  | C11: ACtE; C17:AE | 20.83 | 19 | .35 | 1.00 | .007 | 54.83 |
|  | C11: AE; C17: ACtE | 57.41 | 19 | .00^**^ | .94 | .031 | 91.41 |
|  | C11: ACtE; C17: CtE | 68.21 | 19 | .00^**^ | .92 | .035 | 102.21 |
|  | C11: CtE; C17: ACtE | 84.77 | 19 | .00^**^ | .90 | .041 | 118.77 |

Note: A = additive genetic effects; D = non-additive genetic effects; Ct = twin-shared environmental effects; E = non-shared environmental effects (including measurement error); ACtE model = d=cs=0; AE model = d=cs=ct=0; CtE model = a=d=cs=0; C.D. = cohort differentiation; p = two-sided significance; CFI = Comparative Fit Index; RMSEA = Root Mean Square of Approximation; AIC = Akaike Information Criterion; ** = p < .01 bilateral significance; * = p < .05 bilateral significance
