# Supplementary material for: The genetic and environmental effects on school grades in late childhood and adolescence
Source: PLoS One. 2019 Dec 31;14(12):e0225946. doi: 10.1371/journal.pone.0225946 (PMC6938312; doi:10.1371/journal.pone.0225946)
Supplement: S5 Table — Note. A = additive genetic effects; D = non-additive genetic effects; Ct = twin-shared environmental effects; E = non-shared environmental effects (including measurement error); ACtE model = d = cs = 0; AE model = d = cs = ct = 0; CtE model = a = d = cs = 0; G.D. = group differentiation; sc = twins who were assigned to the same class; ds = twins who were assigned to different classes; p = two-sided significance; ** = p < .01 bilateral significance; * = p < .05 bilateral significance. (DOCX) [file pone.0225946.s005.docx]

**S5 Table. Model comparison: χ2-difference test for models with differentiation between twins who were assigned to the same classroom and twins who were assigned to different classrooms.**

|  | **Model** |  | **χ2** | ***df*** | ***Δ* χ2** | **Δ *df*** | ***p*** |
| --- | --- | --- | --- | --- | --- | --- | --- |
| Mathematics | **ACtE _with G.D._** |  | **19.94** | **18** |  |  |  |
|  |  | ACtE _without G.D._ | 25.00 | 21 | 5.06 | 3 | .17 |
|  |  | sc: ACtE; dc:AE | 22.41 | 19 | 2.47 | 1 | .12 |
|  |  | sc: AE; dc: ACtE | 37.29 | 19 | 17.35 | 1 | .00^**^ |
|  |  | sc: ACtE; dc: CtE | 25.89 | 19 | 5.95 | 1 | .02^*^ |
|  |  | sc: CtE; dc: ACtE | 30.42 | 19 | 10.48 | 1 | .00^**^ |
| German | **ACtE _with G.D._** |  | **28.97** | **18** |  |  |  |
|  |  | ACtE _without G.D._ | 35.70 | 21 | 6.73 | 3 | .08 |
|  |  | sc: ACtE; dc:AE | 29.25 | 19 | 0.29 | 1 | .59 |
|  |  | sc: AE; dc: ACtE | 49.90 | 19 | 20.93 | 1 | .00^**^ |
|  |  | sc: ACtE; dc: CtE | 48.25 | 19 | 19.28 | 1 | .00^**^ |
|  |  | sc: CtE; dc: ACtE | 47.38 | 19 | 18.42 | 1 | .00^**^ |
| GPA | **ACtE _with G.D._** |  | **18.45** | **18** |  |  |  |
|  |  | ACtE _without G.D._ | 32.94 | 21 | 14.49 | 3 | .00^**^ |
|  |  | sc: ACtE; dc:AE | 22.97 | 19 | 4,52 | 1 | .03^*^ |
|  |  | sc: AE; dc: ACtE | 55.08 | 19 | 36.63 | 1 | .00^**^ |
|  |  | sc: ACtE; dc: CtE | 45.77 | 19 | 27.33 | 1 | .00^**^ |
|  |  | sc: CtE; dc: ACtE | 65.80 | 19 | 47.35 | 1 | .00^**^ |

*Note*. A = additive genetic effects; D = non-additive genetic effects; Ct = twin-shared environmental effects; E = non-shared environmental effects (including measurement error); ACtE model = *d*=*cs*=0; AE model = *d*=*cs*=*ct*=0; CtE model = *a*=*d*=*cs*=0; G.D. = group differentiation; sc = twins who were assigned to the same class; ds = twins who were assigned to different classes; *p* = two-sided significance; ** = *p* < .01 bilateral significance; * = *p* < .05 bilateral significance
