# Supplementary material for: The genetic and environmental effects on school grades in late childhood and adolescence
Source: PLoS One. 2019 Dec 31;14(12):e0225946. doi: 10.1371/journal.pone.0225946 (PMC6938312; doi:10.1371/journal.pone.0225946)
Supplement: S6 Table — Note: A = additive genetic effects; D = non-additive genetic effects; Ct = twin-shared environmental effects; E = non-shared environmental effects (including measurement error); ACtE model = d = cs = 0; AE model = d = cs = ct = 0; CtE model = a = d = cs = 0; G.D. = group differentiation; sc = twins who were assigned to the same class; dc = twins who were assigned to different classrooms; p = two-sided significance; CFI = Comparative Fit Index; RMSEA = Root Mean Square of Approximation; AIC = Akaike Information Criterion; ** = p < .01 bilateral significance; * = p < .05 bilateral significance. (DOCX) [file pone.0225946.s006.docx]

**S6 Table. Model comparison tests and fit-statistics for models allowing for differentiation between twins who were assigned to the same classroom and twins who were assigned to different classrooms.**

|  | **Model** | **χ2** | ***df*** | ***P*** | **CFI** | **RMSEA** | **AIC** |
| --- | --- | --- | --- | --- | --- | --- | --- |
| Mathematics | **ACtE _with G.D._** | **19.94** | **18** | **.34** | **.99** | **.014** | **55.94** |
|  | ACtE _without G.D._ | 25.00 | 21 | .25 | .97 | .018 | 55.00 |
|  | sc: ACtE; dc:AE | 22.42 | 19 | .26 | .98 | .018 | 56.41 |
|  | sc: AE; dc: ACtE | 37.29 | 19 | .01^*^ | .88 | .041 | 71.29 |
|  | sc: ACtE; dc: CtE | 25.89 | 19 | .13 | .95 | .025 | 59.89 |
|  | sc: CtE; dc: ACtE | 30.42 | 19 | .05^*^ | .92 | .033 | 64.42 |
| German | **ACtE _with G.D._** | **28.97** | **18** | **.05** | **.95** | **.033** | **64.97** |
|  | ACtE _without G.D_. | 35.70 | 21 | .02^*^ | .93 | .035 | 65.70 |
|  | sc: ACtE; dc:AE | 29.25 | 19 | .06 | .95 | .031 | 63.25 |
|  | sc: AE; dc: ACtE | 49.90 | 19 | .00^**^ | .86 | .054 | 83.90 |
|  | sc: ACtE; dc: CtE | 48.25 | 19 | .00^**^ | .87 | .052 | 82.25 |
|  | sc: CtE; dc: ACtE | 47.38 | 19 | .00^**^ | .87 | .051 | 81.38 |
| GPA | **ACtE _with G.D._** | **28.45** | **18** | **.43** | **1.00** | **.007** | **54.45** |
|  | ACtE _without G.D._ | 32.94 | 21 | .05 | .97 | .032 | 62.94 |
|  | sc: ACtE; dc:AE | 22.97 | 19 | .24 | .99 | .019 | 56.97 |
|  | sc: AE; dc: ACtE | 55.08 | 19 | .00^**^ | .91 | .058 | 89.08 |
|  | sc: ACtE; dc: CtE | 45.77 | 19 | .00^**^ | .93 | .050 | 79.77 |
|  | sc: CtE; dc: ACtE | 65.80 | 19 | .00^**^ | .88 | .066 | 99.80 |

*Note*: A = additive genetic effects; D = non-additive genetic effects; Ct = twin-shared environmental effects; E = non-shared environmental effects (including measurement error); ACtE model = *d*=*cs*=0; AE model = *d*=*cs*=*ct*=0; CtE model = *a*=d=*cs*=0; G.D. = group differentiation; sc = twins who were assigned to the same class; dc = twins who were assigned to different classrooms; *p* = two-sided significance; CFI = Comparative Fit Index; RMSEA = Root Mean Square of Approximation; AIC = Akaike Information Criterion; ** = *p* < .01 bilateral significance; * = *p* < .05 bilateral significance
