# Supplementary material for: The genetic and environmental effects on school grades in late childhood and adolescence
Source: PLoS One. 2019 Dec 31;14(12):e0225946. doi: 10.1371/journal.pone.0225946 (PMC6938312; doi:10.1371/journal.pone.0225946)
Supplement: S7 Table — Note. a2 = additive genetic effects; ct2 = twin-shared environmental effects; e2 = non-shared environmental effects (including measurement error); G.D. = group differentiation. (DOCX) [file pone.0225946.s007.docx]

**S7 Table. Standardized variance components derived from the best fitting model as well as from more parsimonious models.**

|  |  |  | ***a*²** | ***ct*²** | ***e*²** |
| --- | --- | --- | --- | --- | --- |
| Mathematics | ACtE _with G.D._ | Same class | .35 | .35 | .30 |
|  |  | Different classes | .38 | .22 | .40 |
|  | ACtE _without G.D._ | Same class | .34 | .31 | .35 |
|  |  | Different classes | .34 | .31 | .35 |
|  | sc: ACtE; dc: AE | Same class | .35 | .35 | .30 |
|  |  | Different classes | .60 | - | .40 |
| German | ACtE _with G.D._ | Same class | .42 | .37 | .21 |
|  |  | Different classes | .65 | .07 | .28 |
|  | ACtE _without G.D._ | Same class | .47 | .28 | .25 |
|  |  | Different classes | .47 | .28 | .25 |
|  | sc: ACtE; dc: AE | Same class | .42 | .37 | .21 |
|  |  | Different classes | .72 | - | .28 |
| GPA | ACtE _with G.D._ | Same class | .43 | .48 | .09 |
|  |  | Different classes | .60 | .22 | .18 |

Note. a² = additive genetic effects; ct² = twin-shared environmental effects; e² = non-shared environmental effects (including measurement error); G.D. = group differentiation
