# Supplementary material for: The genetic and environmental effects on school grades in late childhood and adolescence
Source: PLoS One. 2019 Dec 31;14(12):e0225946. doi: 10.1371/journal.pone.0225946 (PMC6938312; doi:10.1371/journal.pone.0225946)
Supplement: S8 Table — Note. a = additive genetic effects; ct = twin-shared environmental effects; e = non-shared environmental effects (including measurement error). (DOCX) [file pone.0225946.s008.docx]

**S8 Table. Unstandardized path estimates and 95% confidence intervals for model parameters derived from the best fitting, most parsimonious model.**

|  |  | ***a*** | ***ct*** | ***e*** |
| --- | --- | --- | --- | --- |
| Mathematics | C11 | 0.55 | 0.55 | 0.55 |
|  |  | [0.41 - 0.69] | [0.42 - 0.67] | [0.50 – 0.60] |
|  | C17 | 0.80 | - | 0.67 |
|  |  | [0.72 – 0.87] | - | [0.61 – 0.73] |
| German | C11 | 0.58 | 0.45 | 0.42 |
|  |  | [0.49 – 0.68] | [0.34 – 0.56] | [0.38 – 0.46] |
|  | C17 | 0.51 | 0.42 | 0.58 |
|  |  | [0.36 – 0.66] | [0.25 – 0.58] | [0.52 – 0.63] |
| GPA | C11 | 0.41 | -0.37 | 0.21 |
|  |  | [0.35 – 0.46] | [-0.43 - -0.31] | [0.19 – 0.23] |
|  | C17 | 0.50 | 0.26 | 0.29 |
|  |  | [0.42 – 0.57] | [0.14 – 0.38] | [0.27 – 0.32] |

*Note.* *a* = additive genetic effects; *ct* = twin-shared environmental effects; *e* = non-shared environmental effects (including measurement error)
