# Supplementary material for: The genetic and environmental effects on school grades in late childhood and adolescence
Source: PLoS One. 2019 Dec 31;14(12):e0225946. doi: 10.1371/journal.pone.0225946 (PMC6938312; doi:10.1371/journal.pone.0225946)
Supplement: S9 Table — Note. a = additive genetic effects; ct = twin-shared environmental effects; e = non-shared environmental effects (including measurement error). (DOCX) [file pone.0225946.s009.docx]

**S9 Table. Unstandardized path estimates and 95% confidence intervals for model parameters derived from the best fitting, most parsimonious model.**

|  |  | ***a*** | ***ct*** | ***e*** |
| --- | --- | --- | --- | --- |
| Mathematics | Same class | 0.55 | 0.55 | 0.51 |
|  |  | [0.39 – 0.72] | [0.40 – 0.70] | [0.48 – 0.57] |
|  | Different classes | 0.59 | -0.44 | 0.61 |
|  |  | [0.36 – 0.82] | [-0.70 - -0.18] | [0.52 – 0.69] |
| German | Same class | 0.55 | 0.52 | 0.40 |
|  |  | [0.43 – 0.68] | [0.39 – 0.65] | [0.35 – 0.44] |
|  | Different classes | 0.65 | -0.21 | 0.43 |
|  |  | [0.50 – 0.79] | [-.0.57 – 0.16] | [0.36 – 0.49] |
| GPA | Same class | 0.38 | 0.41 | 0.18 |
|  |  | [0.32 – 0.44] | [0.33 – 0.47] | [0.16 – 0.20] |
|  | Different classes | 0.45 | -0.27 | 0.24 |
|  |  | [0.36 – 0.53] | [-0.40 - -0.14] | [0.20 – 0.28] |

*Note.* *a* = additive genetic effects; *ct* = twin-shared environmental effects; *e* = non-shared environmental effects (including measurement error)
